# A Novel Hyphal Suspension Approach for MIC Testing of Mould Fungi

**DOI:** 10.1111/myc.70177

**Published:** 2026-04-27

**Authors:** Hailin Zheng, Dongmei Li, Naicen Ge, Huan Mei, Xiaofang Li, Weida Liu

**Affiliations:** ^1^ Hospital for Skin Diseases, Institute of Dermatology Chinese Academy of Medical Sciences & Peking Union Medical College Nanjing China; ^2^ Jiangsu Provincial Key Laboratory of Dermatology Nanjing China; ^3^ Department of Microbiology & Immunology Georgetown University Medical Center Washington USA

**Keywords:** dermatophytes, drug susceptibility assay, filamentous fungi, invasive infections, microconidia, mycelial cell, new method, tinea infections

## Abstract

**Background:**

Filamentous fungi cause a wide range of superficial and invasive infections, and antifungal susceptibility assay is essential for guiding effective therapy. Standard broth microdilution methods rely on microconidia‐based inocula; however, many clinically important moulds produce insufficient microconidia due to intrinsic characteristics or culture conditions, limiting the applicability of conventional testing and often necessitating empirical treatment.

**Objectives:**

To overcome this limitation, we developed an alternative method using precisely quantified mycelial cell suspensions for MIC determination.

**Methods:**

The method was evaluated using 115 fungal strains and compared with traditional microconidia‐based testing.

**Results:**

High concordance was observed between the two approaches, with agreement rates of 90% or higher across three common dermatophytes causing tinea infections and three representative fungi responsible for invasive infections.

**Conclusions:**

This mycelial‐based approach provides a reliable and practical alternative for antifungal susceptibility testing of poorly sporulating moulds and may improve the efficiency and accessibility of MIC testing in clinical laboratories.

## 1 Introduction

Filamentous fungi are responsible for a broad spectrum of superficial and invasive infections, including pulmonary aspergillosis, chromoblastomycosis, melanotic sporotrichosis, and various tinea diseases [1]. These pathogens can be broadly categorised into superficial fungi, which cause conditions such as tinea capitis, tinea corporis and tinea pedis [2], and deep or invasive fungi, which may lead to severe or fatal outcomes if not treated promptly [3]. In recent years, both superficial and invasive fungi have developed increasing levels of antifungal resistance, particularly in *Aspergillus fumigatus* , *Trichophyton indotineae* [4, 5]. Moreover, emerging fungal pathogens and species previously considered susceptible—such as *Trichophyton rubrum* and *Microsporum canis*—have also shown newly reported resistance to antifungal agents [6, 7].

To optimise the treatment of fungal infections, especially those caused by drug‐resistant strains, clinicians must rely on antifungal susceptibility testing to guide therapeutic decision‐making [8]. By comparing the susceptibility profiles of different antifungal agents against the same fungal isolate, clinicians can select treatments with the greatest antifungal or fungicidal efficacy while also considering drug availability and cost, thereby improving clinical outcomes [9]. Current fungal drug sensitivity testing using broth microdilution protocols relies primarily on standardised microconidia suspensions [10, 11]. However, many clinically relevant moulds produce insufficient quantities of microconidia [12, 13, 14, 15], either due to intrinsic biological characteristics or limitations of the culture media used. This poses a significant challenge for antifungal susceptibility testing, often resulting in reliance on empirical treatment and potentially leading to therapeutic failure or disease recurrence.

Hyphae constitute the main structural component of filamentous fungi. Addressing the limitations of antifungal susceptibility testing for filamentous fungi is therefore urgent. In this study, we developed an alternative susceptibility testing method based on the preparation of fungal suspensions using accurately quantified mycelial cells rather than microconidia. Although previous studies have explored the use of mycelia for antifungal testing, the results often differed significantly from microconidia‐based methods [16]. These discrepancies are likely due to the excessive length and heterogeneity of hyphae and the lack of precise quantification, as inoculum preparation has commonly relied on spectrophotometric measurements or McFarland standards, which do not accurately reflect the actual number of mycelial units inoculated [17, 18]. To date, no standardised method has been reported for the precise quantification of fungal hyphae.

To overcome the challenges of antifungal susceptibility testing for weakly sporulating or non‐sporulating clinical isolates, there is a critical need for a reliable method to accurately quantify mycelial cells [19]. Precise hyphal quantification would also facilitate a wide range of hypha‐based experimental applications, including gene knockout studies, protein extraction, transcriptomic analyses, fungal–host co‐culture assays, and animal infection models. This approach is particularly valuable for filamentous fungi that produce limited numbers of microconidia.

Here, we report for the first time a method for the precise quantification of mycelial cells and validate its application in antifungal susceptibility testing using representative superficial and invasive filamentous fungi. We anticipate that this work will provide a foundation for the broader clinical and experimental application of mycelial‐based methodologies.

## 2 Materials and Methods

### 2.1 Strains and Growth

A total of 115 clinical fungal isolates were obtained from the Chinese Academy of Medical Sciences Pathogenic Microorganism Species Preservation Center, Medical Fungi sub‐center, which included *T. rubrum* (20), *T*. *mentagrophytes* (18), *M. canis* (20), *Fonsecaea pedrosoi* (17), *Cladophialophora carrionii* (18), *Aspergillus fumigatus* (20), *T*. *mentagrophytes* ATCC4439, and *Trichophyton interdigitale* 05896. The clinical strains were identified based on morphological characteristics and confirmed through sequencing of the conserved ribosomal internal transcribed spacer (ITS) region and large subunit region. All sequences of clinical isolates were submitted to GenBank under accession numbers PX491965–PX492078 for ITS and PX805974–PX806087 for the large subunit.

### 2.2 Antifungal Agents

Fluconazole, ciclopirox, griseofulvin, itraconazole, voriconazole, posaconazole, amorolfine, terbinafine were all purchased from Sigma‐Aldrich Co. LLC (USA). The concentration for fluconazole testing ranged from 0.125 to 64 μg/mL. The concentration for ciclopirox testing ranged from 0.03 to 16 μg/mL. The concentration for griseofulvin testing ranged from 0.015 to 8 μg/mL. The concentration for itraconazole, voriconazole, posaconazole, amorolfine testing ranged from 0.004 to 2 μg/mL. Terbinafine, which ranged from 0.000125 to 0.06 μg/mL, was slightly adjusted based on the CLSI M38‐A2 protocol.

### 2.3 Culture Media

The solid agar culture media used in this study were Potato Dextrose Agar (PDA, OXOID) or potato glucose liquid medium without agar.

### 2.4 Inoculum Preparations

Stock inoculum suspensions were prepared according to the CLSI reference protocol. Briefly, fungal cultures were grown on potato dextrose agar (PDA) slants at 35°C for 3 days for *Aspergillus fumigatus* and for 30°C for 10–14 days for *T. rubrum* , *T*. *mentagrophytes*, *M. canis* , *F*. *pedrosoi* and *C*. *carrionii*. Conidial suspensions were harvested and filtered through a 40 μm pore‐size cell strainer to remove large mycelial aggregates and quantified using a haemocytometer.

For preparation of mycelial inocula, mycelial clumps collected from liquid culture, grown at 30°C on a rotary shaker (220 rpm) for 3–5 days in potato glucose liquid medium, measuring at least 0.5 × 0.5 cm were transferred into a 1.5 mL centrifuge tube containing 1 mL of sterile physiological saline and 2–3 grinding beads. The samples were homogenised using a mechanical grinder until no visible large particles remained. The resulting suspension was then filtered through a 70 μm pore‐size cell strainer to remove large mycelial aggregates. A 10 μL aliquot of the filtrate was loaded onto a haemocytometer for microscopic counting. During counting, only intact mycelial cells exhibiting complete cytoplasm, absence of cytoplasmic leakage, and clear refractive properties under light microscopy were included. Damaged hyphae showing cytoplasmic leakage or lacking refractivity were excluded from quantification. A 70‐μL suspension of *T*. *mentagrophytes* ATCC 4439 hyphal cells (1 × 10^3^ hyphal cells/mL) was spread onto agar plates. After 30°C for 7 days of incubation, colonies were observed. The experiment was repeated three times.

### 2.5 CLSI Broth Microdilution Method

Antifungal susceptibility testing was performed according to the CLSI M38 reference broth microdilution method. The final inoculum concentrations were adjusted to 0.4 × 10^4^ to 5 × 10^4^ CFU/mL for non‐dermatophytes and 1 × 10^3^ to 3 × 10^3^ CFU/mL for dermatophytes in RPMI 1640 medium buffered to pH 7.0 with 0.165 M morpholinepropanesulfonic acid (MOPS). Microdilution panels were incubated at 35°C and examined for visible growth at 48–96 h, depending on the growth rate of each fungal species. Among them, microdilution panels of *M.canis* were incubated at 30°C [16]. Minimum inhibitory concentrations (MICs) were defined as the CLSI M38 reference broth microdilution method.

### 2.6 Data Analysis

All antifungal susceptibility tests were performed in triplicate on different days with also a different culture, and the final MIC value for each isolate was recorded. The geometric mean MIC (GMIC), GMIC_50_ and GMIC_90_ were calculated for each antifungal agent.

The ICC value (intraclass correlation coefficient) is a statistical tool. ICC estimates and their 95% CIs were calculated based on a two‐way mixed‐effects model and agreement on IBM spss statistics software. ICC values were classified as follows: less than 0.50, poor agreement; 0.50–0.74, moderate agreement; 0.75–0.89, good agreement; and 0.90 or greater, excellent agreement [20].

Reproducibility between the microconidia‐based and hyphae‐based methods was assessed by calculating the percentage of essential agreement (EA) between the two sets of MIC values [21]. Essential agreement was defined as MIC values differing by no more than ± two twofold dilutions [22].

## 3 Results

### 3.1 Validation of Hyphal Cell Quantification Method

**FIGURE 1:**
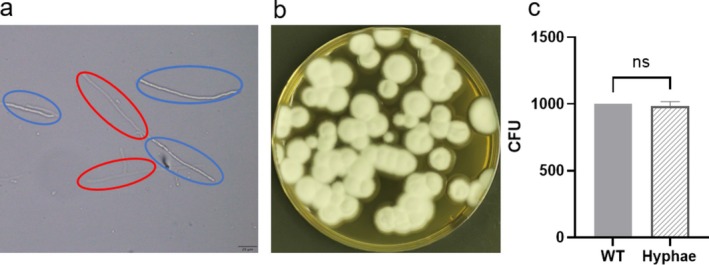
Hyphal cell quantification method. (a) Blue circles indicate intact hyphal cells with normal refractive properties and no cytoplasmic leakage, whereas red circles indicate damaged hyphal cells lacking refractive properties, showing cytoplasmic leakage and mycelial disruption. (b) A 70‐μL suspension of *T*. *mentagrophytes* ATCC 4439 hyphal cells (1 × 10^3^ hyphal cells/mL) was spread onto agar plates. After 7 days of incubation, colonies were observed. (c) Colony‐forming units (CFU) calculated from microscopic counting of 1000 hyphal cells (WT) were compared with the actual colony counts obtained from hyphal cells grown on plates. Statistical analysis using a t‐test showed no significant difference between the two groups.

To validate the feasibility of precise hyphal quantification, a 100 μL conidial suspension of *T*. *mentagrophytes* ATCC 4439 at a concentration of 1 × 10^3^ CFU/mL was inoculated into liquid medium and cultured for 4 days. The resulting mycelial masses were collected, mechanically homogenised, filtered and examined microscopically for counting (Figure 1a). The quantified mycelial suspension was then adjusted to 1 × 10^3^ CFU/mL, and 70 μL aliquots were plated onto PDA plates. Colony counts were recorded after 30°C for 7 days of incubation (Figure 1b). The CFU counts obtained from plate culture were consistent with those derived from microscopic counting, with no significant difference observed between the two methods (Figure 1c).

### 3.2 Microscopic Characterization of Microconidia‐ and Hyphae‐Based Inocula

**FIGURE 2:**
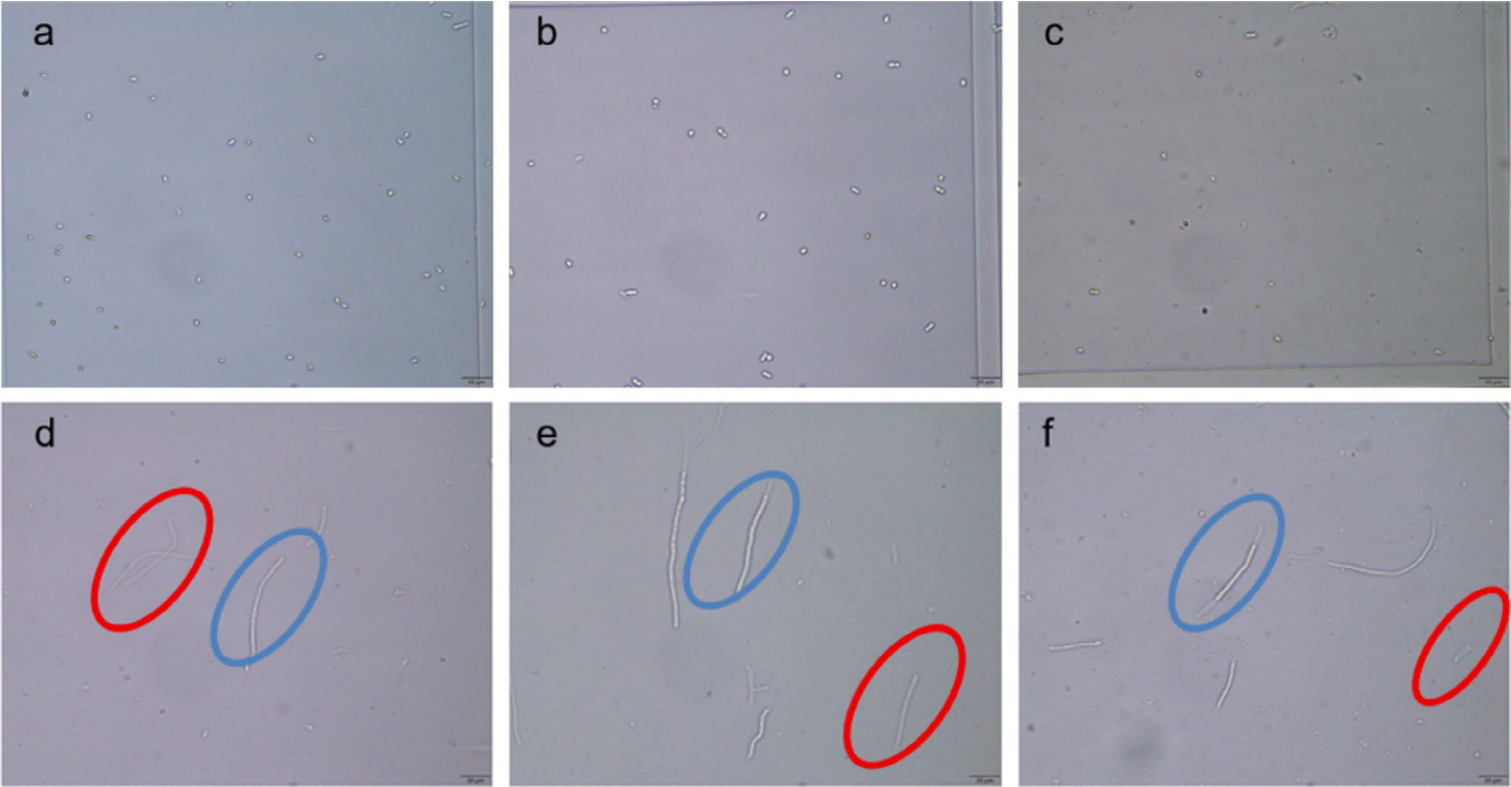
Microscopic comparison of microconidia and hyphae of three superficial fungi. (a) Microconidia of *T. rubrum* 00972; (b) Microconidia of *T*. *mentagrophytes* 01785; (c) Microconidia of *M. canis* 01723; (d) hyphae of *T. rubrum* 00972; (e) hyphae of *T*. *mentagrophytes* 01785; and (f) hyphae of *M. canis* 01723. Blue circles indicate intact cells, whereas red circles indicate damaged hyphal cells.

**FIGURE 3:**
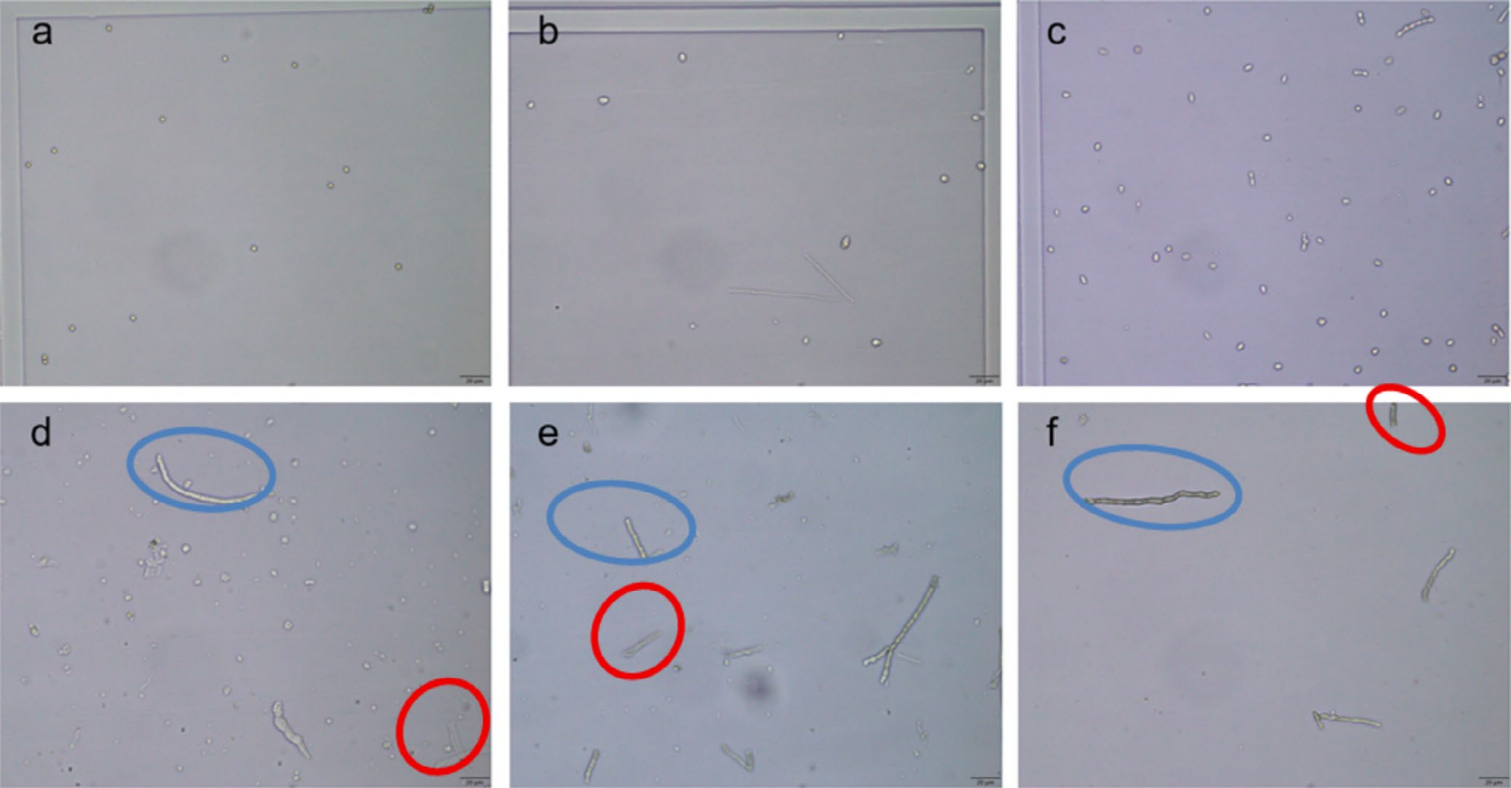
Microscopic comparison of microconidia and hyphae of three deep‐seated fungi. (a) Microconidia of *A. fumigatus* 05090; (b) Microconidia of *F*. *pedrosoi* 04385; (c) Microconidia of *C*. *cladosporioides* 06067; (d) hyphae of *A. fumigatus* 05090; (e) hyphae of *F*. *pedrosoi* 04385; and (f) hyphae of *C*. *cladosporioides* 06067. Blue circles indicate intact cells with normal refractive properties and no cytoplasmic leakage, whereas red circles indicate damaged hyphal cells lacking refractive properties and exhibiting cytoplasmic leakage and mycelial disruption.

Following validation of the hyphal counting method, three common superficial fungi (*T. mentagrop*hytes complex, *T. rubrum* and *M. canis* ) and three representative invasive fungi (*F*. *pedrosoi*, *C*. *carrionii* and *Aspergillus fumigatus* ) were examined microscopically in both microconidia and hyphal forms prior to antifungal susceptibility testing (Figures 2 and 3). Intact fungal cells displaying complete cytoplasm and clear refractive properties were included in inoculum quantification (blue circles), whereas damaged cells lacking refractivity or showing cytoplasmic leakage were excluded (red circles).

### 3.3 Concordance of Antifungal Susceptibility Testing Between Microconidia‐ and Hyphae Based Methods

After separate quantification, equivalent concentrations of microconidia‐based and hyphae‐based inocula were used for broth microdilution susceptibility testing against eight antifungal agents. MIC values were determined according to CLSI criteria, and agreement between the two methods was assessed using essential agreement (±two twofold dilutions; Table 1).

**TABLE 1:** The susceptibility test results of 8 antifungal (Fluconazole, ciclopirox, griseofulvin, itraconazole, voriconazole, posaconazole, amorolfine, terbinafine) drugs for 115 fungal strains.

		Fluconazole	Ciclopirox	Griseofulvin	Itraconazole	Voriconazole	Posaconazole	Amorolfine	Terbinafine
*T. mentagrophytes* complex (20)	Range (A)	8–32	0.25–0.5	0.125–1	0.25–1	0.03–0.06	0.015–0.06	0.015–0.06	0.001–0.008
	MIC50 (A)	8	0.5	0.5	0.5	0.03	0.03	0.03	0.002
	MIC90 (A)	16	0.5	1	1	0.06	0.03	0.06	0.004
	GMIC (A)	11.76	0.41	0.52	0.38	0.036	0.048	0.028	0.002
	Range (B)	2–32	0.125–1	0.125–1	0.06–0.5	0.015–0.06	0.008–0.06	0.015–0.06	0.0005–0.004
	MIC50 (B)	8	0.5	0.5	0.25	0.03	0.03	0.03	0.002
	MIC90 (B)	16	1	1	0.5	0.06	0.03	0.06	0.004
	GMIC (B)	9.70	0.46	0.45	0.24	0.034	0.022	0.025	0.0019
	ICC	0.442	1	0.809	0.577	0.55	0.49	0.478	0.16
	EA (%)	100	100	100	95	100	100	100	100
*T. rubrum* (20)	Range (A)	0.25–16	0.25–0.5	0.25–1	0.125–1	0.004–0.06	0.004–0.25	0.004–0.06	0.00025–0.008
	MIC50 (A)	2	0.25	0.5	0.5	0.03	0.015	0.015	0.002
	MIC90 (A)	2	0.5	1	0.5	0.03	0.125	0.03	0.008
	GMIC (A)	1.36	0.30	0.58	0.41	0.02	0.017	0.014	0.0013
	Range (B)	1–4	0.25–1	0.25–1	0.06–1	0.008–0.06	0.004–0.25	0.004–0.06	0.00025–0.004
	MIC50 (B)	2	0.5	0.5	0.125	0.03	0.015	0.03	0.002
	MIC90 (B)	4	1	1	0.5	0.06	0.03	0.06	0.008
	GMIC (B)	1.53	0.54	0.56	0.19	0.022	0.016	0.021	0.0016
	ICC	0.434	0.019	0.384	0.715	0.699	0.804	0.775	0.86
	EA (%)	100	100	100	95	100	95	100	100
*M. Canis* (20)	Range (A)	32‐ > 64	0.25–1	0.5–4	0.125–0.5	0.06–1	0.06–0.25	0.004–0.06	0.004–0.015
	MIC50 (A)	> 64	0.5	2	0.125	0.5	0.06	0.015	0.008
	MIC90 (A)	> 64	1	4	0.5	1	0.25	0.06	0.015
	GMIC (A)	___	0.46	1.47	0.18	0.32	0.074	0.015	0.01
	Range (B)	32‐ > 64	0.5–2	0.5–1	0.03–0.25	0.03–0.5	0.004–0.06	0.004–0.015	0.002–0.015
	MIC50 (B)	> 64	1	0.5	0.06	0.25	0.015	0.004	0.004
	MIC90 (B)	> 64	1	1	0.25	0.5	0.06	0.015	0.015
	GMIC (B)	___	0.93	0.56	0.054	0.22	0.020	0.006	0.006
	ICC	0.524	0.22	0.037	0.876	0.46	0.729	0.341	0.522
	EA (%)	100	100	90	95	100	90	90	100
*F*. *pedrosoi* (17)	Range (A)	16–64	0.5–2	> 8	> 8	0.25–0.5	0.06–0.25	1‐ > 2	0.03‐ > 0.06
	MIC50 (A)	32	1	> 8	> 8	0.5	0.125	> 2	> 0.06
	MIC90 (A)	32	2	> 8	> 8	0.5	0.25	> 2	> 0.06
	GMIC (A)	27.4	1.17	> 8	> 8	0.45	0.12	___	___
	Range (B)	32–64	1–2	> 8	> 8	0.125–1	0.06–0.25	> 2	0.06‐ > 0.06
	MIC50 (B)	32	2	> 8	> 8	0.5	0.25	> 2	> 0.06
	MIC90 (B)	64	2	> 8	> 8	0.5	0.25	> 2	> 0.06
	GMIC (B)	35.9	1.85	> 8	> 8	0.35	0.16	> 2	___
	ICC	0.186	0.29	1	0.076	0.074	0.451	1	0.316
	EA (%)	100	100	100	100	100	100	100	100
*C*. *carrionii* (18)	Range (A)	16–64	1–2	> 8	0.06–0.5	0.06–0.5	0.015–0.25	2‐ > 2	0.015‐ > 0.06
	MIC50 (A)	16	2	> 8	0.25	0.125	0.125	> 2	0.06
	MIC90 (A)	64	2	> 8	0.5	0.5	0.25	> 2	> 0.06
	GMIC (A)	23.5	1.53	> 8	0.28	0.16	0.13	___	___
	Range (B)	16–64	1–4	> 8	0.125–1	0.125–1	0.03–0.25	2‐ > 2	0.06‐ > 0.06
	MIC50 (B)	64	2	> 8	0.25	0.25	0.125	> 2	> 0.06
	MIC90 (B)	64	4	> 8	0.5	0.5	0.25	> 2	> 0.06
	GMIC (B)	43.55	2.08	> 8	0.34	0.30	0.11	___	___
	ICC	0.746	0.614	1	0.362	0.669	0.409	0.615	0.117
	EA (%)	100	100	100	100	100	100	100	100
*A. fumigatus* (20)	Range (A)	> 64	0.5–2	> 8	0.5‐ > 2	0.25‐ > 2	0.125–1	1‐ > 2	> 0.06
	MIC50 (A)	> 64	0.5	> 8	0.5	0.5	0.25	2	> 0.06
	MIC90 (A)	> 64	2	> 8	1	1	0.5	> 2	> 0.06
	GMIC (A)	> 64	0.58	> 8	___	___	0.29	___	> 0.06
	Range (B)	> 64	1–4	> 8	0.5‐ > 2	0.5‐ > 2	0.125–1	1‐ > 2	> 0.06
	MIC50 (B)	> 64	1	> 8	0.5	0.5	0.25	2	> 0.06
	MIC90 (B)	> 64	4	> 8	1	> 2	0.25	> 2	> 0.06
	GMIC (B)	> 64	1.17	> 8	___	___	0.22	___	> 0.06
	ICC	1	0.889	1	0.951	0.945	0.817	0.479	1
	EA (%)	100	100	100	100	100	100	100	100

*Note:* A:The fungi suspension prepared using fungal microconidia and subjected to drug sensitivity testing. B: The fungi suspension prepared using fungal hyphae and subjected to drug sensitivity testing.

Abbreviations: EA, Essential agreement; ICC, Intraclass Correlation Coefficient.

Among 20 isolates of the *T. mentagrophytes* complex, essential agreement was 95% for itraconazole and 100% for the remaining seven agents. For 20 *T. rubrum* isolates, agreement was 95% for itraconazole and posaconazole and 100% for the other six agents. In *M. canis* , agreement was 90% for griseofulvin, posaconazole, and amorolfine, 95% for itraconazole, and 100% for the remaining four agents. For the dematiaceous fungi *F. pedrosoi* and *C. carrionii*, and for *A. fumigatus* , essential agreement was 100% across all eight antifungal agents tested.

Overall, across six fungal species and 115 isolates, the concordance between microconidia‐based and hyphae‐based antifungal susceptibility testing was consistently high, with essential agreement rates of 90% or greater.

## 4 Discussion

Antifungal susceptibility testing is essential for guiding appropriate clinical therapy, yet current broth microdilution protocols rely primarily on standardised microconidia‐based inocula. Commercially available susceptibility testing systems are largely limited to yeasts and a few moulds, such as *Candida*, *Cryptococcus* and *Aspergillus* species [23]. Many clinically relevant filamentous fungi, however, produce insufficient microconidia due to intrinsic biological characteristics or limitations of culture conditions (23, 24). For instance, nearly half of clinical isolates of *T. rubrum* and *M. canis* exhibit minimal or absent sporulation on PDA [15, 24]. Moreover, the growing identification of rare fungal pathogens—including *T. schoenleinii*, *T. violaceum* , and certain dematiaceous fungi—further complicates susceptibility testing [25, 26]. In such cases, the inability to obtain adequate microconidia often necessitates empirical antifungal treatment, increasing the risk of therapeutic failure or disease recurrence.

To address these limitations, we developed an alternative antifungal susceptibility testing approach based on the preparation of fungal suspensions using accurately quantified mycelial cells rather than microconidia. This method was evaluated using 115 clinical isolates and compared with the conventional microconidia‐based method. The ICC value is commonly used in data analysis to help researchers understand the degree of consistency among different observers or measuring instruments in repeated measurements. In this study, more than half of the ICC values exceeded 0.5, which proved the high consistency of the two methods. At the same time, there were many ICC values that were relatively low. The reason for this was that the drug sensitivity values differed by no more than two twofold dilutions, and all were considered acceptable. However, when calculating the ICC values, the differences were significant. Our results demonstrated a high level of agreement between the two approaches, with concordance rates of 90% or higher across all antifungal agents tested. Importantly, this consistency was observed for both superficial dermatophytes causing tinea infections and representative filamentous fungi responsible for invasive and deep‐seated infections.

The development of standardised susceptibility testing methods for other filamentous fungi has lagged behind, largely because of the difficulty in obtaining precise and quantitative microconidia suspensions. Previous studies attempting to perform antifungal susceptibility testing on weakly sporulating or non‐sporulating fungi often relied on poorly defined inoculum preparation methods, such as crude mycelial grinding followed by sedimentation or optical density measurements. These approaches lacked precise quantification of fungal biomass and frequently resulted in inconsistent or non‐comparable MIC values between mycelial‐ and microconidia‐based methods. In contrast, our study is the first to report a method for precise microscopic quantification of fungal hyphal cells, thereby providing a useful solution for antifungal susceptibility testing of weakly sporulating or non‐sporulating filamentous fungi.

By directly comparing mycelial‐based and microconidia‐based susceptibility testing across six fungal species, we demonstrated that mycelial‐based inoculum preparation is a feasible and reliable alternative for antifungal susceptibility testing when microconidia‐based methods are impractical. Additional advantages of this method include the absence of specialised equipment requirements and relatively low cost, which facilitate its adoption in routine clinical laboratories.

Nevertheless, this method is not universally applicable and is not intended to replace conventional microconidia‐based testing when adequate microconidia are available. For example, it is not suitable for fungi belonging to the order Mucorales, whose sparsely septate hyphae are easily damaged during mechanical processing, resulting in insufficient intact hyphal units for accurate quantification [27]. Fortunately, Mucorales species typically produce abundant microconidia, making conventional microconidia‐based susceptibility testing feasible [28]. For other weakly sporulating or non‐sporulating filamentous fungi encountered in clinical practice, however, we recommend the mycelial counting method as a practical alternative to support accurate antifungal susceptibility testing and facilitate more precise clinical treatment decisions.

## Author Contributions

**Hailin Zheng:** conceptualization, visualization, investigation, funding acquisition, writing – original draft. **Weida Liu:** conceptualization, funding acquisition, resources, supervision. **Xiaofang Li:** conceptualization, funding acquisition, project administration, validation. **Huan Mei:** software, methodology. **Dongmei Li:** writing – review and editing, writing – original draft, methodology. **Naicen Ge:** data curation, formal analysis.

## Funding

This work was supported by The National Natural Science Foundation of China (No. 82402654), The National Key Research and Development Program of China, 2022YFC2504800. The National Science and Technology Infrastructure of China, NPRC‐32. The Scientific and Technological Innovation Projects of Medicine and Health of Chinese Academy of Medical Sciences, 2021‐I2M‐1‐039. Jiangsu Provincial Medical Key Laboratory, Jiangsu Province Capability Improvement Project through Science, Technology and Education, ZDXYS202204.

## Ethics Statement

The authors have nothing to report.

## Conflicts of Interest

The authors declare no conflicts of interest.

## Data Availability

The data that support the findings of this study are available from the corresponding author upon reasonable request.
